# (*S*)-2-Methylpiperazinediium dichloride 0.42-hydrate

**DOI:** 10.1107/S1600536808010519

**Published:** 2008-04-23

**Authors:** William T. A. Harrison

**Affiliations:** a Department of Chemistry, University of Aberdeen, Meston Walk, Aberdeen AB24 3UE, Scotland

## Abstract

The cations and anions of the chiral title compound, C_5_H_14_N_2_^2+^·2Cl^−^·0.42H_2_O, are linked by N—H⋯Cl hydrogen bonds into chains propagating in [100], which contain *R*_4_^2^(14) loops.

## Related literature

For crystal structures containing the same chiral cation, see: Muller *et al.* (2005 ▶); Tuel *et al.* (2002 ▶). For background on graph theory, see: Bernstein *et al.* (1995 ▶).
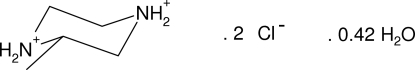


## Experimental

### 

#### Crystal data

C_5_H_14_N_2_^2+^·2Cl^−^·0.42H_2_O
*M* *_r_* = 180.65
Monoclinic, 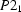

*a* = 5.7548 (2) Å
*b* = 11.6176 (4) Å
*c* = 6.9248 (2) Å
β = 105.7599 (16)°
*V* = 445.57 (3) Å^3^
*Z* = 2
Mo *K*α radiation
μ = 0.66 mm^−1^
*T* = 120 (2) K
0.10 × 0.08 × 0.02 mm

#### Data collection

Nonius KappaCCD diffractometer
Absorption correction: none
5193 measured reflections
1999 independent reflections
1952 reflections with *I* > 2σ(*I*)
*R*_int_ = 0.071

#### Refinement

*R*[*F* ^2^ > 2σ(*F* ^2^)] = 0.042
*wR*(*F* ^2^) = 0.110
*S* = 1.05
1999 reflections
93 parameters
1 restraint
H-atom parameters constrained
Δρ_max_ = 0.93 e Å^−3^
Δρ_min_ = −0.23 e Å^−3^
Absolute structure: Flack (1983 ▶), 929 Friedel pairs
Flack parameter: 0.04 (9)

### 

Data collection: *COLLECT* (Nonius, 1998 ▶); cell refinement: *SCALEPACK* (Otwinowski & Minor, 1997 ▶); data reduction: *DENZO*  (Otwinowski & Minor, 1997 ▶), *SCALEPACK* and *SORTAV* (Blessing, 1995 ▶); program(s) used to solve structure: *SHELXS97* (Sheldrick, 2008 ▶); program(s) used to refine structure: *SHELXL97* (Sheldrick, 2008 ▶); molecular graphics: *ORTEP-3* (Farrugia, 1997 ▶); software used to prepare material for publication: *SHELXL97*.

## Supplementary Material

Crystal structure: contains datablocks I, global. DOI: 10.1107/S1600536808010519/ng2428sup1.cif

Structure factors: contains datablocks I. DOI: 10.1107/S1600536808010519/ng2428Isup2.hkl

Additional supplementary materials:  crystallographic information; 3D view; checkCIF report

## Figures and Tables

**Table 1:** Hydrogen-bond geometry (Å, °)

*D*—H⋯*A*	*D*—H	H⋯*A*	*D*⋯*A*	*D*—H⋯*A*
N1—H2⋯Cl2	0.92	2.25	3.096 (2)	153
N1—H1⋯Cl2^i^	0.92	2.24	3.136 (2)	166
N2—H3⋯Cl1	0.92	2.26	3.149 (2)	163
N2—H4⋯Cl1^i^	0.92	2.30	3.137 (2)	152

Symmetry code: (i) 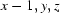.

**Fig. 1.**
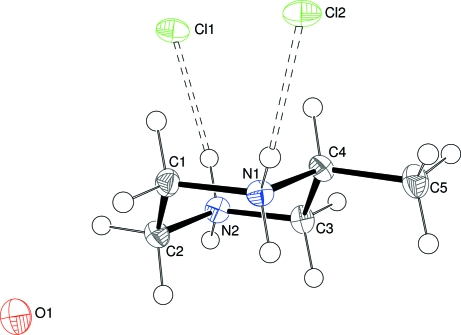
View of the molecular structure of (I) showing 50% displacement ellipsoids (arbitrary spheres for the H atoms). Hydrogen bonds are indicated by double-dashed lines.

**Fig. 2.**
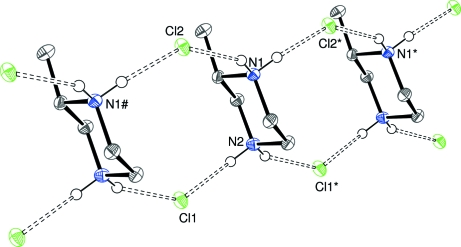
Fragement of a [100] hydrogen bonded chain of cations and anions in the crystal of (I). The carbon-bound H atoms are omitted for clarity. Symmetry code suffixes: (*) x - 1, y, z; (#) x + 1, y, z.

## supplementary crystallographic information

### Comment

The title compound, (I), is a chiral molecular salt, in which the organic species has accepted two protons from the hydrochloric acid. The geometrical parameters of the C_5_H_14_N_2_^2+^ dication (Fig. 1) are similar to those of the same speies in other structures (Muller *et al.*, 2005; Tuel *et al.*, 2002) and its six-membered ring is a typical chair. The C4 stereogenic centre has S configuration and the pendant C5 methyl group occupies an equatorial position with respect to the ring.

In the crystal of (I), the cations and anions are linked by N—H···Cl hydrogen bonds (Table 1) into chains propagating in [100], with two chloride ions bridging each dication, as shown in Fig 2. In terms of graph theory (Bernstein *et al.*, 1995), *R*_4_^2^(14) loops arise from this connectivity.

The O1 water molecule is partially occupied in the crystal of (I), although there is no obvious crystallographic reason (*e.g.* symmetry generated close contacts) as to why this should be the case. Based on short O···Cl contacts of less than 3.5 Å, the water molecule probably participates in O—H···Cl hydrogen bonds thereby helping to crosslink the [100] chains, but the water H atoms could not be found or placed unambiguously in the present study.

### Experimental

Equimolar quantities of 0.1 *M* aqueous (*S*)-2-methylpiperazine and 0.1 *M* aqueous hydrochloric acid were mixed, leading to a clear solution. Colourless plates of (I) grew as the water slowly evaporated.

### Refinement

When refined with full occpancy, atom O1 showed an excessively large *U*_iso_ value of 0.15 Å^2^. Its fractional site occupancy was refined and rapidly converged to 0.420 (11) with a more reasonable *U*_iso_ value and improvement in fit. Its U^ij^ values were subsequently refined and converged without difficulty. Its presumed attached H atoms could not be located from difference maps in the present study. Attempts at geometrical placement were ambiguous, as there are several possible O···Cl contacts that might correspond to O—H···Cl hydrogen bonds.

The other hydrogen atoms were geometrically placed (C—H = 0.95–0.99 Å, N—H = 0.92 Å) and refined as riding with *U*_iso_(H) = 1.2*U*_eq_(C,*N*) or 1.5*U*_eq_(methyl C). The methyl group was allowed to rotate, but not tip, to best fit the electron density.

The highest difference peak is 0.73Å from H1.

### Crystal data

**Table:**

C_5_H_14_N_2_^2+^·2Cl^–^·0.42H_2_O	*F*_000_ = 192
*M**_r_* = 180.65	*D*_x_ = 1.346 Mg m^−^^3^
Monoclinic, *P*2_1_	Mo *K*α radiation λ = 0.71073 Å
Hall symbol: P 2yb	Cell parameters from 2691 reflections
*a* = 5.7548 (2) Å	θ = 2.9–27.5º
*b* = 11.6176 (4) Å	µ = 0.66 mm^−^^1^
*c* = 6.9248 (2) Å	*T* = 120 (2) K
β = 105.7599 (16)º	Plate, colourless
*V* = 445.57 (3) Å^3^	0.10 × 0.08 × 0.02 mm
*Z* = 2	

### Data collection

**Table:**

Nonius KappaCCD diffractometer	1952 reflections with *I* > 2σ(*I*)
Radiation source: fine-focus sealed tube	*R*_int_ = 0.071
Monochromator: graphite	θ_max_ = 27.5º
*T* = 120(2) K	θ_min_ = 3.1º
ω and φ scans	*h* = −7→7
Absorption correction: none	*k* = −14→15
5193 measured reflections	*l* = −8→9
1999 independent reflections	

### Refinement

**Table:**

Refinement on *F*^2^	Hydrogen site location: inferred from neighbouring sites
Least-squares matrix: full	H-atom parameters constrained
*R*[*F*^2^ > 2σ(*F*^2^)] = 0.042	*w* = 1/[σ^2^(*F*_o_^2^) + (0.0558*P*)^2^ + 0.1729*P*] where *P* = (*F*_o_^2^ + 2*F*_c_^2^)/3
*wR*(*F*^2^) = 0.110	(Δ/σ)_max_ < 0.001
*S* = 1.06	Δρ_max_ = 0.93 e Å^−^^3^
1999 reflections	Δρ_min_ = −0.22 e Å^−^^3^
93 parameters	Extinction correction: none
1 restraint	Absolute structure: Flack (1983), 929 Friedel pairs
Primary atom site location: structure-invariant direct methods	Flack parameter: 0.04 (9)
Secondary atom site location: difference Fourier map	

### Special details

**Table:**

Geometry. All e.s.d.'s (except the e.s.d. in the dihedral angle between two l.s. planes) are estimated using the full covariance matrix. The cell e.s.d.'s are taken into account individually in the estimation of e.s.d.'s in distances, angles and torsion angles; correlations between e.s.d.'s in cell parameters are only used when they are defined by crystal symmetry. An approximate (isotropic) treatment of cell e.s.d.'s is used for estimating e.s.d.'s involving l.s. planes.
Refinement. Refinement of *F*^2^ against ALL reflections. The weighted *R*-factor *wR* and goodness of fit *S* are based on *F*^2^, conventional *R*-factors *R* are based on *F*, with *F* set to zero for negative *F*^2^. The threshold expression of *F*^2^ > σ(*F*^2^) is used only for calculating *R*-factors(gt) *etc*. and is not relevant to the choice of reflections for refinement. *R*-factors based on *F*^2^ are statistically about twice as large as those based on *F*, and *R*- factors based on ALL data will be even larger.

### Fractional atomic coordinates and isotropic or equivalent isotropic displacement parameters (Å^2^)

**Table:**

	*x*	*y*	*z*	*U*_iso_*/*U*_eq_	Occ. (<1)
N1	0.7382 (4)	−0.00043 (19)	0.4593 (3)	0.0150 (4)	
H1	0.6241	−0.0130	0.5278	0.018*	
H2	0.8880	−0.0125	0.5468	0.018*	
N2	0.4376 (4)	0.06003 (19)	0.0680 (3)	0.0171 (5)	
H3	0.5504	0.0729	−0.0016	0.020*	
H4	0.2869	0.0723	−0.0181	0.020*	
C1	0.7209 (4)	0.1215 (3)	0.3871 (4)	0.0181 (5)	
H5	0.8509	0.1372	0.3220	0.022*	
H6	0.7418	0.1745	0.5025	0.022*	
C2	0.4781 (5)	0.1420 (2)	0.2391 (4)	0.0190 (5)	
H7	0.3490	0.1324	0.3076	0.023*	
H8	0.4703	0.2219	0.1881	0.023*	
C3	0.4563 (5)	−0.0617 (2)	0.1406 (4)	0.0176 (5)	
H9	0.4345	−0.1146	0.0249	0.021*	
H10	0.3263	−0.0774	0.2058	0.021*	
C4	0.7000 (5)	−0.0841 (2)	0.2891 (4)	0.0164 (5)	
H11	0.8297	−0.0729	0.2197	0.020*	
C5	0.7142 (6)	−0.2064 (3)	0.3697 (5)	0.0228 (6)	
H12	0.8577	−0.2143	0.4839	0.034*	
H13	0.7248	−0.2605	0.2639	0.034*	
H14	0.5694	−0.2232	0.4133	0.034*	
Cl1	0.88946 (10)	0.12630 (6)	−0.08251 (9)	0.02299 (18)	
Cl2	1.28588 (11)	−0.04977 (5)	0.61677 (10)	0.02002 (17)	
O1	0.1426 (11)	0.3549 (5)	0.1162 (10)	0.039 (2)	0.420 (11)

### Atomic displacement parameters (Å^2^)

**Table:**

	*U*^11^	*U*^22^	*U*^33^	*U*^12^	*U*^13^	*U*^23^
N1	0.0128 (10)	0.0182 (10)	0.0123 (11)	0.0011 (7)	0.0003 (8)	0.0017 (8)
N2	0.0175 (10)	0.0190 (11)	0.0128 (11)	0.0002 (8)	0.0010 (9)	0.0012 (8)
C1	0.0173 (12)	0.0148 (12)	0.0210 (13)	−0.0016 (11)	0.0033 (10)	−0.0015 (13)
C2	0.0188 (11)	0.0174 (12)	0.0204 (12)	0.0027 (9)	0.0048 (9)	0.0000 (10)
C3	0.0206 (12)	0.0166 (12)	0.0139 (12)	0.0009 (10)	0.0017 (10)	−0.0010 (10)
C4	0.0180 (11)	0.0165 (11)	0.0146 (12)	0.0031 (9)	0.0042 (9)	−0.0003 (9)
C5	0.0228 (15)	0.0182 (14)	0.0241 (17)	0.0033 (10)	0.0008 (13)	0.0022 (11)
Cl1	0.0175 (3)	0.0339 (4)	0.0176 (3)	0.0027 (3)	0.0048 (2)	0.0015 (3)
Cl2	0.0129 (3)	0.0300 (3)	0.0166 (3)	−0.0009 (2)	0.0031 (2)	−0.0016 (3)
O1	0.042 (4)	0.027 (4)	0.050 (4)	0.004 (2)	0.017 (3)	0.003 (3)

### Geometric parameters (Å, °)

**Table:**

N1—C1	1.497 (4)	C2—H7	0.9900
N1—C4	1.497 (3)	C2—H8	0.9900
N1—H1	0.9200	C3—C4	1.519 (4)
N1—H2	0.9200	C3—H9	0.9900
N2—C2	1.488 (3)	C3—H10	0.9900
N2—C3	1.495 (3)	C4—C5	1.521 (4)
N2—H3	0.9200	C4—H11	1.0000
N2—H4	0.9200	C5—H12	0.9800
C1—C2	1.510 (3)	C5—H13	0.9800
C1—H5	0.9900	C5—H14	0.9800
C1—H6	0.9900		
			
C1—N1—C4	111.7 (2)	N2—C2—H8	109.5
C1—N1—H1	109.3	C1—C2—H8	109.5
C4—N1—H1	109.3	H7—C2—H8	108.1
C1—N1—H2	109.3	N2—C3—C4	111.0 (2)
C4—N1—H2	109.3	N2—C3—H9	109.4
H1—N1—H2	107.9	C4—C3—H9	109.4
C2—N2—C3	110.9 (2)	N2—C3—H10	109.4
C2—N2—H3	109.5	C4—C3—H10	109.4
C3—N2—H3	109.5	H9—C3—H10	108.0
C2—N2—H4	109.5	N1—C4—C3	109.5 (2)
C3—N2—H4	109.5	N1—C4—C5	109.6 (2)
H3—N2—H4	108.1	C3—C4—C5	110.8 (2)
N1—C1—C2	110.0 (2)	N1—C4—H11	109.0
N1—C1—H5	109.7	C3—C4—H11	109.0
C2—C1—H5	109.7	C5—C4—H11	109.0
N1—C1—H6	109.7	C4—C5—H12	109.5
C2—C1—H6	109.7	C4—C5—H13	109.5
H5—C1—H6	108.2	H12—C5—H13	109.5
N2—C2—C1	110.8 (2)	C4—C5—H14	109.5
N2—C2—H7	109.5	H12—C5—H14	109.5
C1—C2—H7	109.5	H13—C5—H14	109.5

### Hydrogen-bond geometry (Å, °)

**Table:**

*D*—H···*A*	*D*—H	H···*A*	*D*···*A*	*D*—H···*A*
N1—H2···Cl2	0.92	2.25	3.096 (2)	153
N1—H1···Cl2^i^	0.92	2.24	3.136 (2)	166
N2—H3···Cl1	0.92	2.26	3.149 (2)	163
N2—H4···Cl1^i^	0.92	2.30	3.137 (2)	152

Symmetry codes: (i) *x*−1, *y*, *z*.
